# Cross-cultural adaptation and validation of the Korean version of the Central Sensitization Inventory in patients undergoing total knee arthroplasty for knee osteoarthritis

**DOI:** 10.1371/journal.pone.0242912

**Published:** 2020-12-01

**Authors:** Man Soo Kim, In Jun Koh, Chul Kyu Kim, Keun Young Choi, Chang Yeon Kim, Yong In

**Affiliations:** 1 Department of Orthopaedic Surgery, Seoul St. Mary’s Hospital, College of Medicine, The Catholic University of Korea, Seoul, Republic of Korea; 2 Department of Orthopaedic Surgery, Eunpyeong St. Mary’s Hospital, College of Medicine, The Catholic University of Korea, Seoul, Republic of Korea; Berner Fachhochschule, SWITZERLAND

## Abstract

The purpose of this study was to establish a Korean version of the Central Sensitization Inventory (CSI-K) for Korean-speaking patients facing total knee arthroplasty (TKA) for knee osteoarthritis (OA) and to investigate the psychometric characteristics of the CSI-K. We recruited a total of 269 patients with knee OA who were scheduled to undergo TKA for the study. CSI-K and pain-related outcomes, including the pain visual analog scale (VAS) and the Western Ontario and McMaster Universities OA Index (WOMAC) pain sub-scores, were measured. Since central sensitization (CS) is closely related to the quality of life (QOL) and limited functionality as well as pain, the patient’s function was measured using the WOMAC function sub-scores, and QOL was measured using the EuroQol five-dimension test (EQ-5D). Reliability and validity were evaluated. Exploratory factor analysis (EFA) was conducted to begin the data reduction to validate the existing questionnaire translation. The internal consistency was excellent, with a Cronbach's alpha of 0.941. The test-retest reliability was acceptable-to-excellent with an ICC of 0.888. As expected, the CSI scores correlated strongly with the WOMAC pain scores (*r* = 0.524, *p* < 0.001) and moderately with the pain VAS (*r* = 0.496, *p* < 0.001), the WOMAC function (*r* = 0.408, *p* < 0.001), and the EQ-5D scores (*r* = 0.437, *p* < 0.001). EFA resulted in a six-factor model. The findings demonstrate that the CSI was successfully trans-culturally adapted into a simplified Korean version (CSI-K) that was reliable and valid for Korean-speaking patients who awaiting TKA for knee OA.

## Introduction

Total knee arthroplasty (TKA) is the most effective surgical method for treating the pain caused by chronic knee osteoarthritis (OA) [1, 2]. A growing number of patients have undergone TKAs for knee OA [3]. The number of TKAs performed in Korea over the last decade has increased significantly, comparable to that reported in some Western countries [4]. Therefore, questionnaires that could evaluate patients who underwent TKA for knee OA are always the subject of attention and interest [1]. Persistent pain patterns have become a major social issue for patients with knee OA as well as for those who have undergone TKA for pain from knee OA [5–7]. No particular reasons have been reported for the symptoms of patients complaining of persistent pain, even though the pain source was removed by the TKA procedure [7, 8]. However, the concept of central sensitization (CS) has recently emerged [7–9].

CS is defined as an abnormal and intense increase in pain caused by mechanisms in the central nervous system (CNS) [10]. CS-related pain was shown to be caused by the increased excitability of the dorsal horn by nociceptive stimuli [10]. This was characterized by hyperalgesia and allodynia [10–12]. CS has been introduced as a cause of pain in various chronic musculoskeletal disorders, including OA [13–15], fibromyalgia [14, 16], rheumatoid arthritis [17], chronic low back pain [14, 18], and chronic patellar tendinopathy [19]. Among these, pain caused by OA related to CS has been the subject of much recent research and attention [11, 12]. In the general population, about 10% of the persistent physical symptoms could not be explained by a clear cause [20]. Among the patients with knee OA, about 20–30% reported persistent pain despite treatment [5, 6], and 5–10% of the patients still complained of persistent pain after TKA [7, 21]. This persistent pattern of pain for unknown reasons demonstrated the need for a screening test for CS. Screening for CS by the Central Sensitization Inventory (CSI) was reported to provide more active and adequate treatment and appropriate education and information for the patients [9, 22, 23].

CS-related research has been actively carried out on TKA as well as on knee OA [9, 11, 12, 14]. The preoperative screening of centrally sensitized patients was shown to be important because CS was associated with persistent pain and dissatisfaction following TKA for knee OA [9, 11, 13, 23, 24]. In the past, CS was diagnosed using quantitative sensory testing (QST). However, QST is limited in clinical practice because it requires time and resources to perform the tests [25]. Thus, the CSI was developed, which could easily be administered as a questionnaire without being invasive [24]. In fact, the CSI has been validated in many countries [24, 26–30]. In Korea, however, there has been no validation study of the CSI, especially among TKA candidates for knee OA. Therefore, our purpose was to establish a Korean version of the CSI (CSI-K) for Korean-speaking Korean patients facing TKA with knee OA and investigate the psychometric characteristics of the CSI-K.

## Methods

All procedures performed in the studies involving human participants were in accordance with the ethical standards of the institutional and/or national research committee and with the 1964 Declaration of Helsinki and its later amendments or comparable ethical standards. This study was approved by the Institutional Review Board of our institution (Seoul St. Mary’s Hospital, The Catholic University of Korea). Study number was KC19RISI0894. All patients provided informed consent.

### Translation procedure

The Korean translation was performed according to the instructions provided by Guillemin et al. [31] using the cross-cultural adaptation process. This process not only ensured the proper translation of the language but also was adapted to maintain the validity of the content throughout the culture. The cross-cultural adaptation process was carried out in six steps: translation, synthesis, reverse translation, expert committee review, pretesting, and evaluation submission. In short, the English version of the CSI was translated separately by two Korean bilingual interpreters. After reaching a uniform agreement between the two translators, a pre-test Korean-translation version was established. This version was originally translated by two bilingual, native English speakers who were blinded to the English version. We continued this process until any disagreements between the English and Korean versions were resolved. When the consensus version was formed, a reverse-translated English version was sent and approved by the Korean Knee Society members. The final edition was pretested in 20 Korean patients with knee OA.

### Study participants

The first 282 TKA candidates with end-stage knee OA were asked to participate in this study. The exclusion criteria included patients diagnosed with primary neuropathic pain (e.g., painful diabetic polyneuropathy), congenital deformity, history of previous knee infection, history of surgery on the ipsilateral or contralateral leg, cancer, dementia, poor Korean-language comprehension, and those who declined to participate in the study. Thirteen patients were excluded, including two with primary neuropathic pain, one with a congenital deformity, three with previous surgery history, five with cancer, and two with dementia. Finally, 269 patients facing TKA due to primary knee OA were enrolled in the final analyses. All patients underwent TKA at our hospital from March to October 2019.

### Procedure

All patient demographics including age, sex, operation side, height, weight, and body mass index (BMI) were recorded. Prospectively, the preoperative CSI-K and pain-related outcomes, including the pain visual analog scale (VAS) and the Western Ontario and McMaster Universities OA Index (WOMAC) [32] pain sub-scores, were evaluated in all patients awaiting TKA. Many studies have been conducted on the relationship between CS and pain, function, and the quality of life (QOL) in OA patients [9, 22, 33–37]. Since CS is closely related to QOL [33, 34, 36, 37] and limited functionality [9, 37] as well as pain [9, 22, 33, 35], function was measured using the WOMAC function sub-scores [32] and QOL was measured using the EuroQol five-dimension test (EQ-5D) [38].

The CSI is a newly developed and validated self-reported inventory to assess patients with central sensitivity syndrome (CSS) [23, 24]. The CSI is divided into two sections, parts A and B. Part A consists of a 25-item questionnaire about physical and emotional symptoms, including headaches, fatigue, sleep disorders, cognitive disorders, and psychological disorders often observed in CS, and questions about pain sensitivity in daily life, including waking unrefreshed in the morning, stiff and achy muscles, anxiety attacks, grinding or clenching teeth, diarrhea and/or constipation, needing help to perform daily activities, sensitivity to bright lights, being easily tired by physical activity, pain all over the body, headaches, feeling discomfort or burning during urination, poor sleep quality, difficulty in concentrating, skin problems, stress that makes the physical symptoms worse, sadness or depression, low energy, muscle tension in the neck and shoulders, pain in the jaw, dizziness and nausea caused by certain smells, frequent urination, uncomfortable and restless legs, poor memory, childhood trauma, and pelvic pain [23, 24].

Scores were not measured in part B, which was used to determine the presence of one or more specific disorders, such as restless leg syndrome, chronic fatigue syndrome, fibromyalgia, temporomandibular joint disorder, migraine or tension headaches, irritable bowel syndrome, multiple chemical sensitivities, or neck injuries. Each item was rated on a 5-point Likert scale from 0 to 4 (0 = not at all, 1 = almost, 2 = sometimes, 3 = often, 4 = always). The CSI scale ranged from 0–100, with 0 being the worst score and 100 being the best score [23, 24]. The CSI score was classified into five CSI severity subgroups of increasing severity: subclinical (0–29), mild (30–39), moderate (40–49), severe (50–59), and extreme (60–100) [35]. Neblett et al. [35] suggested that a CSI score of 40 points was the cutoff value to confirm CS. Therefore, we used a CSI score of 40 as the criterion for dividing patients into high and low CSI score groups.

The WOMAC has been widely used as an indicator for evaluating knee OA and TKA patients [32] and has already been translated and validated for Korean patients. The WOMAC is a self-managed questionnaire that includes 24 questions classified into three subscales of pain, stiffness, and physical function. Each question is scored on a 5-point Likert scale format as none (0), mild (1), moderate (2), severe (3), and extreme (4). The score for each subscale is calculated by summing the component item scores for each subscale. There are five questions on pain, two on stiffness, and 14 on function. Thus, the possible score ranges are 0–20 for pain, 0–8 for stiffness, and 0–68 for physical function, and the final total aggregate scores range from 0 to 96 points.

The EQ-5D is a measurement tool commonly used for health-related quality of life. It was developed by the Euro Quality of Life (EuroQol) Group [38, 39]. It consists of five categories: mobility, self-care, usual activities, pain/discomfort, and anxiety/depression. Each category is measured by three grades; no problem, some problems, and extreme problems. In addition, one visual index value measures health status. The biggest strength of this tool is that it is very simple and the subjects can easily respond to the statements. The EQ 5D also had a validated and translated Korean version for evaluating QOL [38, 39]. The Korean versions of the WOMAC and EQ-5D have been widely used in Korea, and their reliability, validity, and responsiveness have been rigorously tested [32, 38].

### Statistical analysis

#### Reliability

Reliability was evaluated by internal consistency and test-retest reliability. Internal consistency was measured using Cronbach’s alpha. A Cronbach’s alpha of 0.5–0.6 was evaluated as poor, one between 0.60 and 0.70 was considered acceptable, between 0.70 and 0.90 was considered good, and higher than 0.90 was excellent [40]. The test-retest reliability was evaluated by the intra-class correlation coefficient (ICC), using a two-way mixed-effects model for absolute agreement. All patients completed the questionnaires four weeks apart without any intervening treatment. The ICC was considered to reflect good reliability at values between 0.40 and 0.75 and excellent reliability at values over 0.75 [41].

#### Validity

To find the concurrent validity, we assessed the domains of the CSI-K by comparing them to the appropriate subscales of the Korean WOMAC and the Korean EQ-5D using Spearman’s coefficients. The correlation was considered strong if the value was greater than 0.5, moderate if the value was between 0.5 and 0.35, and weak if the value was less than 0.35 [42].

#### Factor analysis

Exploratory factor analysis (EFA) was conducted to begin data reduction to validate the existing questionnaire translations using the maximum-likelihood method with Promax rotation. In the EFA, the number of dimensions was measured as eigenvalues, which should be 1.0. In addition, the correlation and contribution of a single item in a dimension can be evaluated using the loading factor, and to contribute enough to the dimension, the cutoff for loading should be set at 0.4 [29, 30].

Statistical analyses were performed using SPSS ver. 21.0 (SPSS, Inc., Chicago, IL, USA). The mean values are shown with the standard deviation (SD), and a *p* < 0.05 or less was taken to indicate significance.

## Results

### Participants

Table 1 contains the baseline characteristics of the participants including the demographic and clinical profiles. There were no missing data. In total, the mean score of the CSI-K was 33.4 ± 15.7.

**Table 1:** Patient demographics and characteristics*.

	Mean (SD) or N (%)
Demographic data	
Age (years)	70.7 (7.7)
Gender (female)	236 (87.7)
Height (cm)	155.3 (7.9)
Weight (Kg)	64.3 (11.3)
BMI (kg/m^2^)	26.6 (3.7)
Operation side (Right)	148 (55.0)
CSI Score	33.4 (15.7)
Pain VAS score	6.2 (1.6)
WOMAC pain score	11.8 (4.7)
WOMAC function score	45.3 (14.5)
Health-related QOL (EQ-5D)	9.0 (1.7)

* Data are presented as mean (standard deviation) or number (percentage).

BMI, body mass index; CSI, Central Sensitization Inventory; VAS, visual analog scale; WOMAC, Western Ontario and McMaster Universities OA Index; QOL, quality of life; EQ-5D, EuroQol five-dimension test.

### Reliability

The internal consistency was excellent, with a Cronbach's alpha of 0.941. The Cronbach’s alphas for the individual CSI items ranged from 0.739 to 0.920. The test-retest reliability was acceptable-to-excellent with ICCs of 0.888 (95% confidential Interval [CI]: 0.860–0.911). Sixteen of the 25 items of the CSI had an excellent ICC greater than 0.75, and nine of the 25 items showed good reliability, with a range from 0.586 to 0.748 (Table 2).

**Table 2:** Cronbach’s alpha, intraclass correlation coefficients, and 95% confidence intervals (lower-upper boundaries) of test-retest reliability.

Item	Question	Cronbach alpha	Intraclass Correlation Coefficients	95% Confidence Intervals (Lower-Upper Boundaries)
Sum	Total Score	0.941	0.888	0.860–0.911
**1**	Unrefreshed in morning	0.876	0.779	0.727–0.822
**2**	Muscles stiff/achy	0.917	0.847	0.809–0.877
**3**	Anxiety attacks	0.862	0.758	0.702–0.804
**4**	Grind/clench teeth	0.798	0.664	0.591–0.726
**5**	Diarrhea/constipation	0.888	0.798	0.750–0.837
**6**	Need help daily activity	0.859	0.753	0.821–0.889
**7**	Sensitive to bright lights	0.856	0.748	0.690–0.796
**8**	Easily tired w/physical activity	0.861	0.756	0.700–0.803
**9**	Pain all over body	0.852	0.742	0.684–0.792
**10**	Headaches	0.835	0.716	0.652–0.770
**11**	Bladder/urination pain	0.902	0.821	0.778–0.857
**12**	Do not sleep well	0.858	0.752	0.695–0.799
**13**	Difficulty concentrating	0.808	0.678	0.608–0.738
**14**	Skin problems	0.884	0.792	0.743–0.833
**15**	Stress makes symptoms worse	0.886	0.795	0.747–0.835
**16**	Sad or depressed	0.871	0.772	0.719–0.816
**17**	Low energy	0.886	0.795	0.746–0.835
**18**	Tension in neck and shoulder	0.920	0.851	0.815–0.881
**19**	Pain in jaw	0.739	0.586	0.501–0.659
**20**	Certain smells make me dizzy	0.864	0.761	0.706–0.807
**21**	Urinate frequently	0.849	0.738	0.678–0.788
**22**	Restless legs	0.866	0.763	0.708–0.809
**23**	Poor memory	0.834	0.715	0.651–0.769
**24**	Trauma as child	0.806	0.675	0.605–0.735
**25**	Pelvic pain	0.856	0.748	0.690–0.796

### Validity

As expected, the CSI scores strongly correlated with the WOMAC pain scores (*r* = 0.524, *p* < 0.001) and moderately with the pain VAS (*r* = 0.496, *p* < 0.001), the WOMAC function (*r* = 0.408, *p* < 0.001), and the EQ-5D scores (*r* = 0.437, *p* < 0.001).

### Factor analysis

We used EFA to evaluate whether the original English CSI study dimensionality and factor-loading patterns were like the Korean subject sample. The contributing factors with item loadings higher than 0.4 are shown in Table 3. The Kaiser-Meyer-Olkin value (0.861) and significant Bartlett’s test of sphericity (*p* < 0.001) indicated that factor analysis was appropriate for this sample. EFA resulted in a six-factor model. Four factors showed behavior similar to that of the original English version items, including physical symptoms, emotional distress, urological symptoms, and headache/jaw symptoms. Items 12 to 17 were loaded on factor 1, which was named “Emotional Distress.” Factor 2 consisted of items 1, 2, 3, 6, 9, 22, and 23, and was named “Physical Symptoms.” Factor 3 consisted of items 1, 7, 10, and 19, and was named “Headache and Jaw Symptoms.” Factor 4 consisted of items 5, 8, 18, 21, and 25, and was named “Urological Symptoms.” Factor 5 consisted of items 4, 14, and 20, and was named “High Central Sensitivity” and items 6 and 24 were loaded on factor 6, which was named “Psychiatric Problems.” Factor 1, Emotional Distress, contained four items (items 13, 15, 16, and 17) relating to emotional distress in the original article [24]. Items 3, 23, and 24 did not affect factor 1 in this study. Items 12 and 14 were included in factor 1 in this study. These items were in the category of physical symptoms in the original English [24] and Serbian versions [28]. Factor 2, which was named Physical Symptoms in this study, included four items, 2, 6, 9, and 22, which related to physical symptoms on the original English version [24]. In this study, items 8, 12, and 18 did not affect factor 2. Items 1, 3, and 23 were included in factor 2 in this study. These items were included in emotional distress in the original English [24] and Brazilian versions [26]. Factor 3 in this study represented included three related items (7, 10, 19) related to headache/jaw symptoms in the original study [24]. Factor 4, named Urological Symptoms, contained two items (21 and 25) related to urinary symptoms in the original study [24]. Items 5, 8, and 18 included in factor 4 in this study were associated with physical symptom categories [24, 28]. In factor 5, items 4 and 20 corresponded to high central sensitivity in the Dutch version [29] and headache/jaw symptoms in the original English version [24]. Item 24, suffering caused by childhood trauma, included in factor 6, was a general category of emotional distress, but it was a psychiatric symptom of trauma that went beyond the usual emotional aspect, which indicated an association with the “requirement for assistance in daily activity” (Item 6). Item 6 included in factor 6 was also included in factor 1. The factor loading of item 11 was < 0.40 (Table 3). The inter-correlations between the factors are presented in Table 4. All inter-factor correlation coefficients were positive after oblique PROMAX rotation.

**Table 3:** CSI factor analysis of the specific CSI items contributing to each factor.

Item	Question	Factor 1	Factor 2	Factor 3	Factor 4	Factor 5	Factor 6	Items not loading
**1**	Unrefreshed in morning		0.447	0.680				
**2**	Muscles stiff/achy		0.768					
**3**	Anxiety attacks		0.649					
**4**	Grind/clench teeth					0.783		
**5**	Diarrhea/Constipation				0.856			
**6**	Need help daily activity		0.635				0.512	
**7**	Sensitive to bright lights			0.652				
**8**	Easily tired w/ physical activity				0.418			
**9**	Pain all over body		0.403					
**10**	Headaches			0.755				
**11**	Bladder/urination pain							X
**12**	Do not sleep well	0.692						
**13**	Difficulty concentrating	0.696						
**14**	Skin problems	0.462				0.436		
**15**	Stress makes symptoms worse	0.759						
**16**	Sad or depressed	0.836						
**17**	Low energy	0.657						
**18**	Tension in neck and shoulder				0.411			
**19**	Pain in jaw			0.695				
**20**	Certain smells make me dizzy					0.702		
**21**	Urinate frequently				0.863			
**22**	Restless legs		0.506					
**23**	Poor memory		0.594					
**24**	Trauma as child						0.872	
**25**	Pelvic pain				0.574			

CSI, Central Sensitization Inventory.

**Table 4:** PROMAX factor correlations of the Korean CSI in patients.

	Factor 1	Factor 2	Factor 3	Factor 4	Factor 5	Factor 6
**Factor 1**	-					
**Factor 2**	0.441	-				
**Factor 3**	0.330	0.436	-			
**Factor 4**	0.417	0.256	0.338	-		
**Factor 5**	0.303	0.080	0.164	0.223	-	
**Factor 6**	0.313	0.081	0.260	0.195	0.186	-

CSI, Central Sensitization Inventory.

The CSI scores among the 269 patients ranged from 0 to 93, with a mean of 33.4 (SD = 15.7). The proportion of patients with different CSI severities and the number of patients in each subgroup were: subclinical (36.1%, *n* = 97); mild (31.2%, *n* = 84); moderate (21.5%, *n* = 58); severe (7.1%, *n* = 19); and extreme (4.1%, *n* = 11). Eighty-eight patients (32.7%) had a CSI score of over 40, indicating CS (Table 5).

**Table 5:** Prevalence of CS severity levels and frequency of diagnoses.

	N (%)
CSI-K score	
**Subclinical (0–29)**	97 (36.1)
**Mild (30–39)**	84 (31.2)
**Moderate (40–49)**	58 (21.5)
**Severe (50–59)**	19 (7.1)
**Extreme (> 60)**	11 (4.1)
**Diagnoses**	
**Restless leg syndrome**	10 (3.7)
**Chronic fatigue syndrome**	10 (3.7)
**Fibromyalgia**	5 (1.9)
**Temporomandibular joint disorder**	6 (2.2)
**Migraine or tension headaches**	21 (7.8)
**Irritable bowel syndrome**	13 (4.8)
**Multiple chemical sensitivities**	2 (0.7)
**Neck injury (including whiplash)**	16 (5.9)
**Anxiety or panic attacks**	8 (3.0)
**Depression**	14 (5.2)

CS, Central Sensitization; CSI-K. Korean version of Central Sensitization Inventory.

## Discussion

The aim of this study was to verify and confirm the basic structure of the CSI-K in a sample of Korean patients who were awaiting TKA for knee OA. Our results showed that the CSI-K had good internal consistency and test-retest reliability. In addition, it showed positive relationships with the pain VAS scores, and the WOMAC pain and function sub-scores, indicating validity of the results. There was also a significant negative correlation between the CSI-K scores and the EQ-5D scores. Factor analysis showed that the CSI-K had a six-factor structure, in contrast to the English [24], Dutch [29], Japanese [30], Brazilian [26], and Serbian [28] versions.

Cronbach's alpha for internal consistency was 0.9 for the CSI-K used in this study. The 25 sub-items also showed high Cronbach alpha values of 0.7 to 0.9. The excellent internal consistency was comparable to that in the Japanese [30], Dutch [29], American [24], and Serbian versions [28], which were 0.89, 0.91, 0.879, and 0.909, respectively. The high Cronbach alphas in most cultures, as well as in Korea, indicated that the CSI was stable in the other forms.

The ICC indicated very good test-retest reliability in our study, at 0.941. The results were similar to 0.85 in the Japanese version (one week), 0.91 in the Brazilian version (two weeks), 0.88 in the Dutch version [29] (five days), 0.817 in the American version (five days), and 0.947 in the Serbian version [28] (one week). In all of the aforementioned countries, the test-retest reliability of the CSI was excellent. The time interval between the test and retest ranged from five days to two weeks. Our study took this into account, with a one-week gap between the first and second tests. Because of the re-measurement within a short period of time, the change in clinical manifestations appeared to be relatively low, indicating a higher ICC.

The CSI-K demonstrated adequate construct validity when compared to the Korean pain VAS, the Korean WOMAC, and the Korean EQ-5D. The correlation tests showed strong a correlation with the pain subscales of the Korean WOMAC and moderate correlation with its function subscales. Pain and functional limitations caused by CS also had a significant effect on the QOL of the patients. Therefore, it would be very important to understand the relationship between the CSI and QOL in patients with TKA for knee OA. In the current study, the CSI-K showed a moderate positive correlation with QOL measured by the EQ-5D (*r* = 0.437, *p* < 0.001) [22, 33]. Our study demonstrated similar levels of correlation as the Japanese version of the CSI [30], perhaps because of the similar cultural backgrounds of the Asian Korean and Japanese populations [43]

The average score of the CSI-K was 33 points in this study. The average Japanese score was 21.9 points [30], the American was 52.4 points [24], the Dutch was 43.8 points [29], and the Brazilian average score was 45.4 points [26]. The CSI-K score was lower than that in Western countries [24, 26, 29], and higher than that in Japan [30]. If the CSI score was over 40, it was considered to be in the high CSI score group, indicating CS [35]. In this study, 33% of the patients had a CSI score of over 40. The proportion of CS patients with a CSI score of over 40 was 10% in the Japanese study [30], 44.2% in the Serbian study [28], and 61.1% in the Brazilian study [26]. The difference in the CSI scores and the proportion of CS patients in each country resulted not only from the cultural differences but also from the differences in the characteristics of the patient populations [24, 26, 28–30]. In this study, CSI was examined only in patients undergoing TKA because of knee OA. Other studies, however, included patients with chronic pain that included OA as well as other disorders [24, 26, 28–30]. In this study, the proportion of CS patients showed a ratio similar to that of studies that examined CSI in existing knee-OA patients [9, 23]. CS could be a risk factor for persistent pain [11], dissatisfaction after TKA [9], and postoperative wound complications [23]. Therefore, it would be important to provide proper information to the patients by means of preoperative education.

There were numerous limitations to this study that should be acknowledged. First, our subjects were recruited from one hospital, which could limit generalization elsewhere in Korea. Second, the majority of the patients were elderly women, which should be taken into account as a demographic feature of TKAs in Korea [44–47]. Because of the lack of male patients, further research would be essential to investigate these factors in Korean men. Third, the sample size was limited and might not have represented the entire Korean population with knee OA. In addition, patients with knee OA who received conservative treatment or had hip OA were not included in this study. It would be necessary to validate the CSI-K and evaluate its responsiveness in patients with knee OA and hip OA who received conservative treatment. Fourth, the CSI was assessed using a patient self-reported questionnaire, which potentially included response bias. Finally, although the method of translation was rigorous and repetitive, some inconsistencies might have remained in the translation from one language to another. If better words or phrases are suggested, they should be validated and tested using the same standardized protocol.

## Conclusion

The CSI-K was successfully trans-culturally adapted into a simplified Korean version that was demonstrated to be reliable and valid in Korean-speaking patients who awaiting TKA for knee OA. In addition, we found evidence for the convergent validity of the CSI-K by its association with pain, function, and the QOL in patients with TKA for knee OA. This scale is a reliable instrument for evaluating the CS of Korean patients with knee OA and is useful for psychometric measurements in clinical research.
